# Tunable Optical and Multiferroic Properties of Zirconium and Dysprosium Substituted Bismuth Ferrite Thin Films

**DOI:** 10.3390/molecules27217565

**Published:** 2022-11-04

**Authors:** A. Sathiya Priya, D. Geetha, J. M. Siqueiros, Ștefan Ţălu

**Affiliations:** 1 Department of Physics, Sri Sairam Engineering College, Chennai 600044, Tamil Nadu, India; 2 Department of Applied Sciences and Humanities, Anna University, MIT Campus, Chennai 600044, Tamil Nadu, India; 3 Centro de Nanociencias y Nanotecnologia, Universidad Nacional Autonoma de México, Km 107 Carretera Tijuana-Ensenada, Ensenada 22860, Baja California, Mexico; 4 The Directorate of Research, Development and Innovation Management (DMCDI), Technical University of Cluj-Napoca, Constantin Daicoviciu St., No. 15, 400020 Cluj-Napoca, Cluj County, Romania

**Keywords:** doped BFO thin films, non-centrosymmetric, solar cells, spin coating

## Abstract

This work presents optical and multiferroic properties of bismuth ferrite thin films that are affected by zirconium and dysprosium substitution. Non-centrosymmetric BiFeO_3_,Bi_0.95_Zr_0.05_FeO_3_, and Bi_0.95_Dy_0.05_FeO_3_ thin films were coated on Pt/TiO_2_/SiO_2_/Si substrates using the spin coating method. The crystal structure, optical properties, microstructural, ferromagnetic, and ferroelectric properties of doped bismuth ferrite thin films were systematically investigated. From the XRD patterns, all the prepared thin films matched well with the rhombohedral structure with *R3c* space group with no observed impurity phases. The average crystallite size of the bismuth ferrite thin films were between 35 and 47 nm, and the size depended on the type of dopant. The determined energy band gap values of BiFeO_3_, Bi_0.95_Dy_0.05_FeO_3_, and Bi_0.95_Zr_0.05_FeO_3_ thin films were 2.32 eV, 2.3 eV, and 2 eV, respectively. Doping of Dy and Zr at the Bi site led to reduced surface roughness. The prepared thin films exhibited enhanced ferromagnetic and ferroelectric properties. The remnant magnetization of Zr-doped BiFeO_3_ was greater than that of the BiFeO_3_ and Dy-doped BiFeO_3_ thin films. From the obtained results, it was concluded that Zr-doped BiFeO_3_ thin films are suitable for solar cell fabrication.

## 1. Introduction

Non-centrosymmetric multiferroic materials have extensive applications in different kinds of fields, such as photovoltaic cells, photocatalytic degradation, random access memory devices, photodetectors, detection of missiles, secure space-to-space communications, and spintronic devices [1,2,3,4,5]. In particular, non-centrosymmetric ferroelectric materials are promising materials for applications in modern renewable energy systems, such as solar cell technology. Hence, many researchers give special attention to their study and production. This study is focused on the ferroelectric and ferromagnetic properties of multiferroic material. The magnetic character is usually driven by exchange interactions between magnetic dipoles associated with unfilled shells of electron orbitals. The electric property, on the other hand, is the result of the ordering of local electric dipoles associated with lone pair electrons. The simultaneous presence of ferromagnetic and ferroelectric properties is an exciting feature that can be applied to information storage, processing, and transmission. Ferroelectric thin films have potential applications in many fields, but some of them do not fill the requirements for commercialization; some of the issues being large leakage current, low nanoscale polarization value, low piezoelectric coefficient, and poor thermal stability. Correspondingly, ferromagnetic thin films are applicable to sensors, magnetic storage, and magnetoresistance elements. Ferromagnetic thin films also suffer from large leakage currents that can be prevented through the preparation method and by properly doping the parent materials. Suppressing oxygen vacancies reduces leakage current. In summary, control or elimination of the above-mentioned problems of ferroelectric and ferromagnetic thin films will potentiate commercial applications.

Bismuth ferrite is a unique non-centrosymmetric multiferroic compound that has attracted great interest due to its remarkable properties and potential applications. It is a perovskite material exhibiting multiferroic properties, such as ferroelectricity and magnetic response at room temperature, owing to its high Curie temperature (T_C_ ~ 850 °C) and Neel temperature (T_N_ ~ 370 °C) [6]. It displays different functional properties, such as ferroelectricity [7,8,9], magnetism [10,11] optical properties [12,13], dielectric susceptibility, and piezoelectricity [14], among others. Based on those properties, it has great potential for technological applications in nonvolatile memory and photovoltaic devices. However, it has a large leakage current that has hindered its technological possibilities. Here, we propose a strategy to overcome the drawbacks and enhance its ferroelectric and ferromagnetic properties.

Doping is a fruitful source of different physical and chemical properties [15,16,17]. For instance, the modification of the ferroelectric and magnetic properties of transition metal- and rare earth-doped BiFeO_3_ is well documented in previous reports [18,19,20,21,22]. Holmium-doped BiFeO_3_ samples showed good ferroelectric performance as compared to undoped ones [23]. Similarly, Nd-doped BiFeO_3_ thin films for random access memory applications were successfully grown [24]. Kathirvel et al. [25] reported that Zr doped in Fe site BiFeO_3_ thin films had enhanced magnetic properties. Liu et al. reported multi-element (La, Er, Zn, Ti) doping of BiFeO_3_ thin films, resulting in reduced leakage current and improved ferroelectric properties [26]. Ni-doped BiFeO_3_ nanoparticles displayed reduced optical band gap compared to undoped BiFeO_3_ nanoparticles [27]. The appropriate substitutions and optimized concentrations are key parameters to appreciably modify the properties of a material. Here, BiFeO_3_ thin films are fabricated using the spin coating method. This technique has advantages such as a simple set up, fast deposition rates, and the ability to work at room temperature and pressure. Deposition can be performed with different kind of substrates and shapes; it is a low-cost procedure and a relatively simple deposition method. This research aims to investigate the potential of bismuth ferrite thin films as candidates for efficient photovoltaic solar cells, energy harvesting, and memory device applications. Doping with Zr and Dy will be tested, aiming to enhance the structural, morphological, ferroelectric, magnetic, and optical properties of bismuth ferrite thin films. The objective of this manuscript is threefold: viz: (a) to deposit films of Zr- and Dy-doped BiFeO_3_ using the spin coating method (b), to describe the mechanism of Zr- and Dy-doped BiFeO_3_ thin films deposition, and (c) to study the structural, optical, morphologic, ferroelectric, and ferromagnetic features of the Zr- and Dy-doped BiFeO_3_ thin films.

## 2. Experimental Methods

### 2.1. Preparation of Precursor Solution

**Figure 1:**
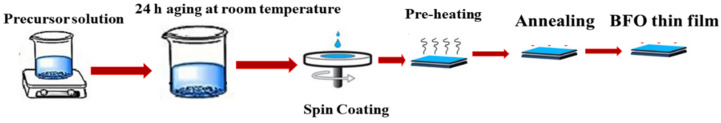
Pictographic representation of fabrication of BFO thin films assembly.

The preparation of the precursor solutions for the sol-gel method goes as follows: stoichiometric amounts of bismuth nitrate (Bi(NO_3_)_3_·5H_2_O (99%)), iron nitrate (Fe(NO_3_)_3_·5H_2_O (98%)), and zirconium nitrate (Zr(NO_3_)_3_·6H_2_O (99%) were dissolved in acetic acid (CH_3_COOH (99%)). Ethylene glycol ((CH_2_OH)_2_ (98%)) was added as a thickening agent. Figure 1 shows the sol-gel synthesis of BiFeO_3_ precursor solutions. Stoichiometric amounts of bismuth nitrate and ferric nitrate were dissolved in distilled water with dilute nitric acid (3 ml), and acetic acid was added to the mixture. Aqueous solutions of Bi_0.95_Zr_0.05_FeO_3_ in molar proportions were prepared. Excess Bi (10 % mol) was added to the mixture to compensate for bismuth loss during the heat treatment. The solution was kept under stirring at 90 °C for 3 h. During the heating process, the solution slowly turned into a viscous brown gel. A similar preparation method was followed for Dy-doped BFO solutions with dysprosium nitrate instead of zirconium nitrate. In what follows, the symbols BFO, BZFO, and BDFO are used for BiFeO_3_, Bi_0.95_Zr_0.05_FeO_3_, and Bi_0.95_Dy_0.05_FeO_3,_ respectively.

### 2.2. Device Fabrication

The Pt/TiO_2_/TiO/SiO_2_/Si substrates were subjected to ultrasonic cleaning in acetone, ethanol, and distilled water for 15 min each. The substrate was dried in a nitrogen gas atmosphere. Figure 1 shows the spin-coating method of BiFeO_3_ thin films. The BFO, BDFO, and BZFO solutions were spin-coated on a Pt/TiO_2_/SiO_2_/Si substrate at 3000 rpm for 30 s. The deposited wet thin films were placed on a hot plate at 150 °C for 4 min and preheated at 500 °C for 10 min. Using consecutive heating processes, multiple layers of thin films were annealed at 500 °C for 1 h in a nitrogen (N_2_) atmosphere. Furthermore, a semitransparent-square-shaped Au electrode (500 μm × 500 μm) with a thickness of approximately 43 nm was deposited on the prepared BFO-based thin films through a shadow mask using vacuum thermal evaporation [28,29].

The crystal structure of the BFO, BDFO, and BZFO thin films was determined using a X-ray diffractometer (XRD, D8 Advanced Bruker, Karlsruhe, Germany) with a Cu Kα radiation of wavelength 1.5404 Ả at the scanning rate of 0.02 min^−1^ for 2 h. The UV-Visible absorption studies were measured using a UV-Vis spectrometer (UV-3600 Plus, Shimadzu, Japan) in a wavelength range from 300 nm to 700 nm. The ferroelectric characteristics were determined using a LC ferroelectric tester (Radiant Technology). The ferromagnetic properties of the prepared BFO thin films were measured using a Lake Shore 7404 vibrating sample magnetometer (VSM, Lake Shore, Westerville, OH, Franklin, USA). The surface morphology of the BFO thin films was measured using field emission scanning electron microscopy (FESEM; Zeiss Sigma-500, Oberkochen, Germany). The surface micromorphology was analyzed using an Atomic Force Microscopy (AFM: NT-MDT, Solver Nano, Moscow, Russia) in the tapping mode at room temperature (23 ± 1 °C) to obtain 256 × 256 pixel images. The silicon nitride tips were used to acquire 3-D surface topography information on scanning areas of 5 × 5 μm^2^ on the samples [30].

## 3. Results and Discussion

### 3.1. Structural Analysis

**Figure 2:**
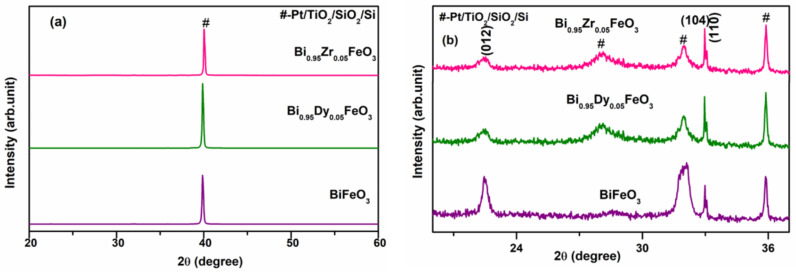
(**a**) XRD pattern of BFO, BDFO, and BZFO thin films (2θ range is 20° to 60°); (**b**) XRD pattern of BFO, BDFO, and BZFO thin films (2θ range is 20° to 38°).

The XRD studies shown in Figure 2 confirmed the perovskite structure of the pristine and doped BFO thin films. The observed XRD patterns of all the prepared thin films were indexed as a rhombohedral structure belonging to the R3c space group. 

Figure 1 reveals the prepared thin films are crystalline in nature. From the Debye–Scherrer theory, the relative integrated intensity of the diffracted beams is given by the following formula [31]:

(1) $$I={\left|F\right|}^{2}P\left(\frac{1+\cos^{2}2\theta }{\sin^{2}2\theta \cdot \cos\theta }\right)$$

where *I* is the relative integrated intensity (arbitrary units), F is the structure factor, p is the multiplicity factor, and *θ* is the Bragg angle. Here, only the factor F was considered in this measurement, as the other factors contributed weakly. The volume fractions of (100), (110), and (111) oriented grains were determined from the integrated intensity of each diffraction peak. The volume fractions of BFO of the (012), (104), and (110) oriented grains were 12.3%, 70.4%, and 17.1%, respectively. Those of the BZFO were 4.1%, 22.4%, and 73.5%, respectively. The intensity of the (012) BDFO and BZFO peaks appears weak compared to the corresponding BFO peak due to the Bi deficiency. These values are instrumental to explain why BZFO and BDFO thin films have better ferroelectric properties than undoped BFO thin films [32].

The average crystallite size of the prepared thin film was determined from Scherrer’s equation [31]:

(2) $$L=\frac{k_{s}\cdot \lambda }{\left(\cos\theta \right)\tau }$$

where *L* is the average size of the ordered (crystalline) domains, *k_s_* is the shape factor constant, *λ* is the X-ray wavelength, *τ* is the peak width at full width half maximum (FWHM), and *θ* is the Bragg angle. The average crystallite sizes of BFO, BDFO, and BZFO were determined to be 36.2 nm, 43.1 nm, and 47 nm, respectively, indicating that doping—in this case with Dy and Zr—led to grain growth in BFO thin films. Furthermore, the dislocation density determined using formula δ = 1/D^2^ also affected particle size. As seen in Table 1, the dislocation density was low for BDFO and BZFO as compared to that of BFO. The lattice parameters were determined using the UnitCellWin software, whereas bond lengths were calculated using VESTA software.

Table 1 shows the lattice parameters and bond lengths for BFO, BDFO, and BZFO thin films. The structure stability of the perovskite structure of the prepared undoped and doped thin films was determined through Goldschmidt’s tolerance factor [33]:

(3) $$t=\frac{r_{A}+r_{O}}{\sqrt{2\left(r_{B}+r_{O}\right)}}$$

where *r_A_* is the average ionic radius of Bi, *r_B_* is the average ionic radius of Fe cations, and *r_O_* is the ionic radius of the oxygen anion. The tolerance factor values of the prepared BFO thin films are shown in Table 1. It turns out that the calculated values are smaller than one, indicating that the thin films possess a distorted rhombohedral structure.

### 3.2. Optical Study

**Figure 3:**
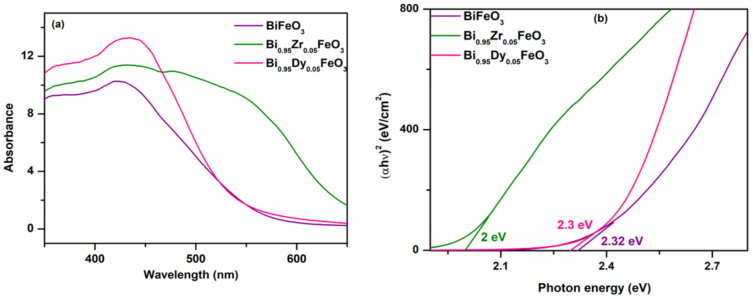
(**a**) Absorbance spectra of BFO, BDFO, and BZFO thin films, (**b**) plot of (αhʋ)^2^ versus hʋ for BFO, BDFO, and BZFO thin films.

Figure 3 shows the UV-Vis absorption spectra of BFO, BDFO, and BZFO thin films taken at room temperature. The corresponding absorption band edge of the BFO, BDFO, and BZFO thin films were found at 421 nm, 426 nm, and 434 nm, respectively. Fe-O band charge transfer, for instance, arose at around 434 nm. The absorption band at 468 nm was associated with the electronic transitions from valence band O2p states to conduction band Fe 3d states. In general, Figure 3 helps to explain how selective doping influences the optical absorption of BFO thin films. The absorption of BDFO and BZFO thin films in the visible region of the spectrum is stronger than that of BFO thin films, a fact that is indicative of their potential applications in solar cell technology. Figure 3b shows Tauc plots, that is, curves of (αhʋ)^2^ versus photon energy for the direct bandgap BFO. The plots provide the experimental values for BFO, BDFO, and BZFO thin films, which are 2.32 eV, 2.3 eV, and 2.0 eV, respectively. The energy band gap value of BZFO is the lowest compared to the other prepared thin films and is associated with a higher absorption capability. A good way to tune the energy bandgap is to vary the concentrations of Fe, Zr, and Dy in the Bi site. It is to be expected that the enhanced absorption property of BZFO thin films will improve the performance of BFO solar cells. The photogenerated excitons play an important role in modifying the photo physical properties of non-centrosymmetric materials. The smaller band gap values of the BZFO thin films produce higher absorption of visible light, which is good for solar cells applications. Combined with the change in the remnant polarization value of BFO, it was found that the optical bandgap decreases as the remnant polarization value increases (according to Section 3.5).

### 3.3. Surface Morphology and Domain Structure Analysis

**Figure 4:**
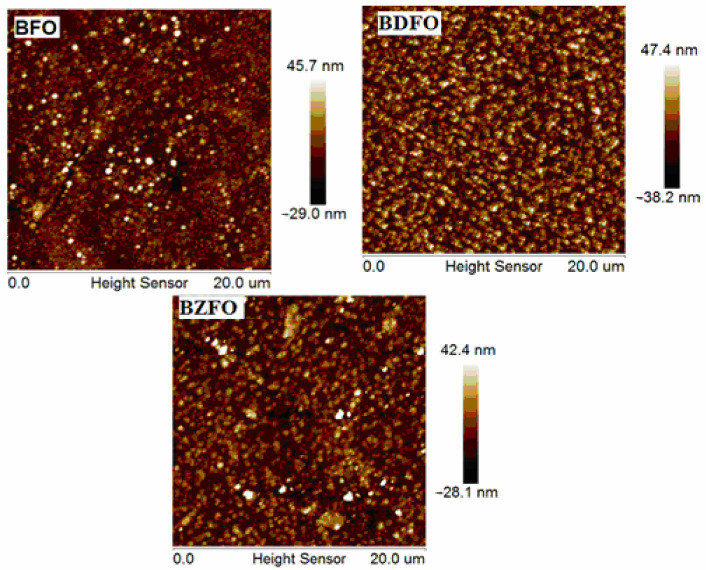
Surface topography images of BFO, BDFO, and BZFO thin films.

**Figure 5:**
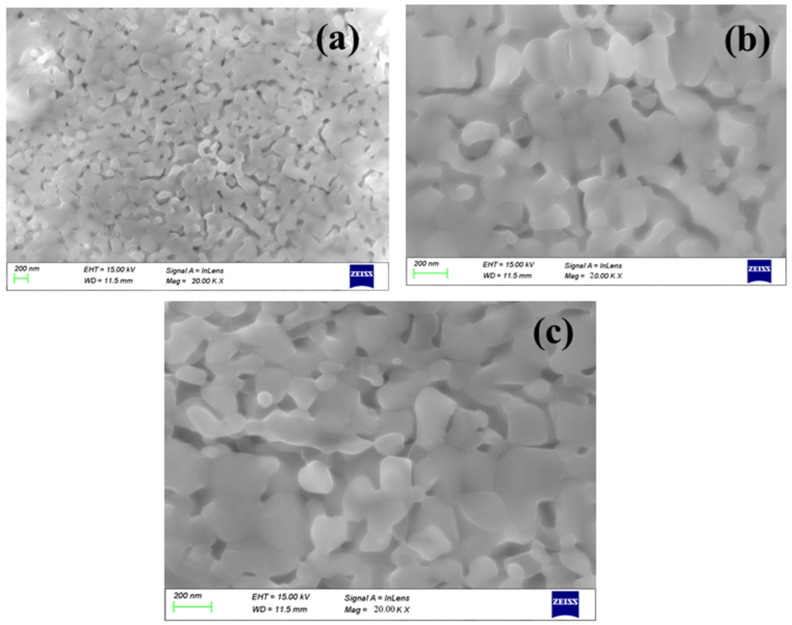
SEM morphology of: (**a**) BFO; (**b**) BDFO; and (**c**) BZFO thin films.

Figure 4 shows the surface topography images of BFO, BDFO, and BZFO thin films determined by AFM. The BFO thin film surface has an uneven surface, which is unfavorable for the properties of the thin films. However, BDFO and BZFO thin films exhibit rough surfaces with dense microstructure. Dy and Zr doping was found to be beneficial to improve the microstructure of the thin films. The values of the root mean square surface roughness were determined for BFO, BDFO, and BZFO thin films to be 91.94, 83.03, and 75.34 nm, respectively. The prepared thin films show crack-free and uniform microstructure, as shown in Figure 4. The homogeneous microstructure has an effect on the ferroelectric properties and will be discussed in Section 3.5 [34]. Figure 5 shows the surface morphology features of the BFO, BDFO, and BZFO thin films. Large pores were observed in the BFO thin films. In BDFO and BZFO thin films, flower-like morphology features were observed from SEM micrographs. The average grain size was also calculated from the SEM study and was about 21.55 (±11.42) nm, 34.68 (±11.42) nm, and 44.30 (±11.42) nm for BFO, BDFO, and BZFO, respectively. Grain size increased in the doped BFO thin films due to Zr and Dy doping. BZFO showed a larger average grain size compared to the other studied thin films. Grain sizes affected the remnant polarization, as explained in Section 3.4 [34].

### 3.4. Ferromagnetic Analysis

**Figure 6:**
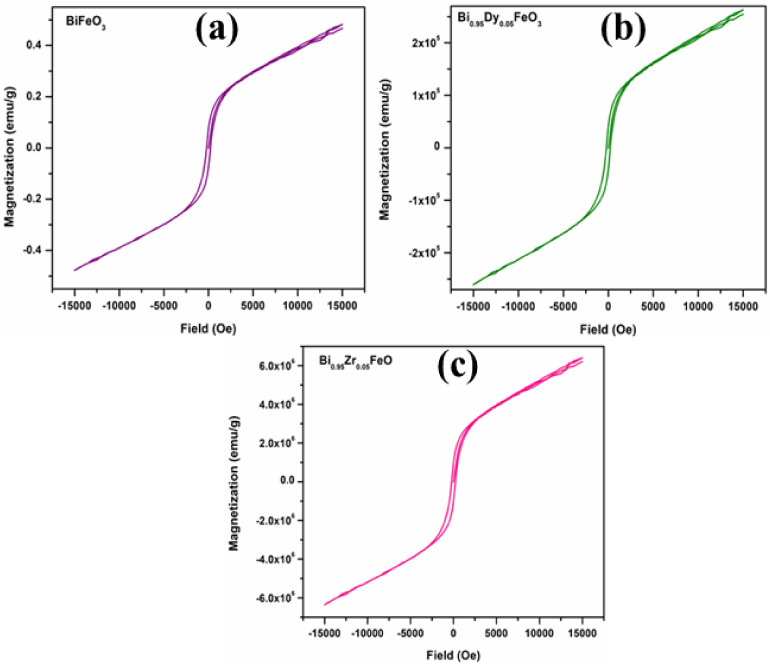
Magnetic hysteresis loops of: (**a**) BFO, (**b**) BDFO, and (**c**) BZFO thin films.

Figure 6 displays the magnetic hysteresis loops for BFO, BDFO, and BZFO thin films, showing slim, well-saturated hysteresis loops characteristic of ferromagnetic behavior. Table 2 summarizes the saturation magnetization, remnant magnetization, and coercive values for the BFO, BDFO, and BZFO thin films. The table shows that doped BFO has a large coercive field compared to undoped BFO thin films. From the displayed values, it is concluded that the magnetization depends on the doping element.

**Figure 7:**
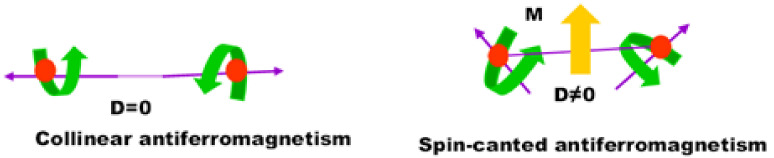
Canted antiferromagnetism spin configuration causes net magnetization in doped bismuth ferrite thin film by the Dzyaloshinskii–Moriya interaction.

The small saturation magnetization values of undoped BFO thin films can be attributed to the spin modulation spiral structure, which obstructs the observation of net magnetization [33,35]. The doped BDFO and BZFO thin films showed much stronger magnetization compared with undoped BFO thin films. The values of the remnant magnetization of BFO, BDFO, and BZFO thin films are displayed in Table 2. The enhanced magnetic properties in BZFO thin films can be attributed to a combination of a change in the spin structure of BFO plus a long-range spiral modulation, and to the fact that the ferromagnetic properties of thin films are enhanced for particles with sizes smaller than 62 nm [36]. That is, particle size influences the ferromagnetic properties due to the destruction of the long wavelength period of the magnetization of bismuth ferrite thin film leaving uncompensated spins which are measurable [37]. In bismuth ferrite, first-principles calculations showed that the Fe-O-Fe bond angle is the critical parameter in the transition from the anti-ferromagnetic phase to the ferromagnetic phase. The tilts of the Fe ions spins are related to the Dzyaloshinskii–Moriya (DM) interaction energy of the Fe ions (H_DM_) [38]:

(4) $$H_{DM}=\sum_{n=0}^{n}\vec{D_{n}}\cdot \left({\vec{S}}_{0}\times {\vec{S}}_{n}\right)$$

where $\vec{D_{n}}=V_{0}\cdot \left[\vec{r_{n-0}}\times \vec{r_{n-n}}\right]$ is the interaction parameter of DM interaction, $\vec{r_{n-0}}$, $\vec{r_{n-n}}$ are the position vectors of the nearest neighbor magnetic ions, from the *n*th O ions to the nearest magnetic Fe ions, and *V*_0_ is the microscopic constant. $\vec{S_{n}}$ are the vectors of the two Fe ions magnetic moments. In the perovskite structure, the Fe-O-Fe bond angle is 180°; therefore, the value of the $\left[\left[\vec{r_{n-0}}\times \vec{r_{n-n}}\right]r_{n-0}\right]$ factor is zero. From that condition, it follows that the anti-symmetric exchange energy term H_DM_ value is also zero. H_DM_ increases when φ begins to depart from the ideality of 180°, as shown in Figure 7. Hence, it is estimated that the magnetization value will increase. It has been shown that the magnetization is sensitive to small changes in lattice constants [39] and that they also participate in improving the ferromagnetic coupling. Sati et al. [40] reported very weak ferromagnetism in Bi_0.95_Dy_0.05_FeO_3_ ceramics, and the reported values are shown in Table 3.

M. Arora et al. [41] reported well saturated ferromagnetism but a low value of saturation magnetization, coercive field, and remnant magnetization in Zr-doped BiFeO_3_ nanoparticles. C. Lan et al. [42] reported low remnant magnetization and weak ferromagnetism in Bi_0.9_La_0.1_Fe_0.98_Zr_0.02_O_3_. In turn, J. Wei et al. [43] reported weak ferromagnetism and low saturation magnetization values in BiFe_0.95_Zr_0.05_O_3_ BiFe_0.90_Zr_0.10_O_3_ thin films, which are presented in Table 3. In conclusion, when compared to previous reports by other authors, BDFO and BZFO came up with better ferromagnetic properties, establishing them as a better choice for memory device applications.

### 3.5. Ferroelectric Analysis

**Figure 8:**
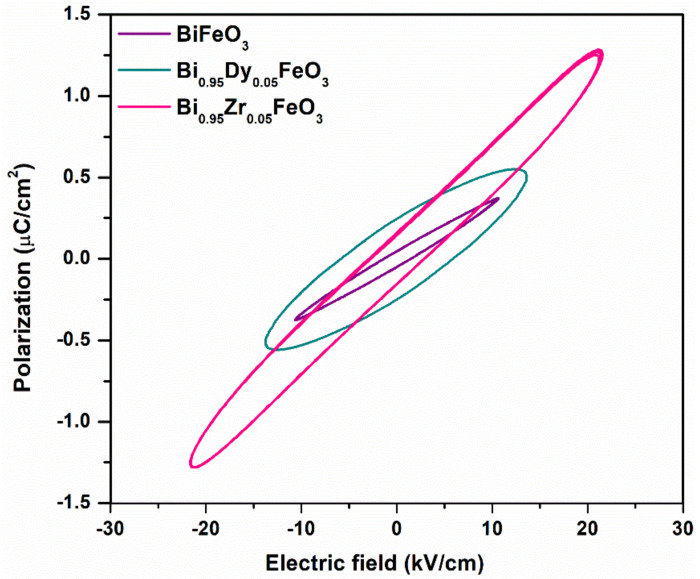
Room temperature P-E hysteresis loops for BFO, BDFO, and BZFO thin films.

The polarization versus electric field hysteresis loops for the BFO, BDFO, and BZFO thin films are shown in Figure 8. In general, in a given non-centrosymmetric sample, the polarization can be reoriented by an external electrical field in the pyroelectric state. This phenomenon is called ferroelectricity. A small volume with electric dipoles pointing in the same direction in the ferroelectric material is a ferroelectric domain. The ferroelectric character of undoped and doped BFO is discussed in what follows. It can be seen that doped BFO thin films present better ferroelectric properties when compared to undoped BFO thin films. The BFO thin films have lower remnant polarization due to oxygen vacancies that can bring about microstructure distortion of the perovskite unit cell and suppress the shift of the B-site. Doping with appropriate elements, such as Dy and Zr, is a very effective way to enhance the ferroelectric properties of BFO thin films. The values of the remnant polarization and coercive field of BFO, BDFO, and BZFO thin films are displayed in Table 4. BZFO, for instance, has a large remnant polarization and a lower coercive field. This is consistent with the surface topography of the films shown in Figure 4. It is evident from the figure that the homogeneity of the film increases with the addition of dopants, i.e., BDFO and BZFO films possessing reduced surface roughness and homogeneous structure exhibit improved ferroelectric characteristics. The increase in remnant polarization can be attributed to inhomogeneity. Hence, homogeneous microstructure has an effect on the ferroelectric properties [47]. Grain size is calculated from SEM in Section 3.3. It is seen that size affects domains and remnant polarization. Defect dipoles are also beneficial for improving polarization, and larger average grain size leads to easier flipping of ferroelectric domains, thus improving the remnant polarization [34]. BDFO and BZFO thin films have better ferroelectric properties, consistent with the structural analysis in Section 3.1. Our reported BDFO ferroelectric P-E loop shows better characteristics than that of Yan et al. [45]. Moreover, the energy bandgap also participates in the ferroelectric properties. The electrons in the valence band are excited to the conduction band by absorbing photons with energies larger than the bandgap.

In general, perovskite samples behave as good insulators, but here, we observed that non-centrosymmetric perovskites act similar to semiconductor materials, as seen from a UV-visible spectrum study. Furthermore, the maximum photon energy of solar radiation is within the wavelength of visible and infrared region. Here, the energy bandgap value of the prepared materials is in the 2–2.32 eV interval. Therefore, if the goal is to prepare high performance solar cells, the studied non-centrosymmetric perovskite materials seem appropriate because of their smaller band gaps.

## 4. Conclusions

Non-centrosymmetric BFO, BDFO, and BZFO thin films were coated on Pt/TiO_2_/SiO_2_/Si substrates using the spin coating method. The impacts on the microstructure, ferromagnetic behavior, and ferroelectric behavior of Dy and Zr substitution have been systematically analyzed. The obtained results encourage us to expect a better photovoltaic response in these non-centrosymmetric thin films based on the combined effects of reduction in the energy gap and grain characteristics. From the structural analysis, the perovskite structure of the thin films without impurity phases was confirmed. Ferromagnetic M-H hysteresis loops demonstrated that the BZFO films display better remnant magnetization values compared to the other studied thin films. In particular, higher saturation magnetization of BZFO thin films compared to BDFO and BFO thin film was observed. A high saturation magnetization value was found in the doped samples and is attributed to the canting of the Fe ions spins, the Fe^3+^-O-Fe^2+^ exchange interaction, and the destruction of the spiral spin cycloid at the interface layer in the prepared film. BZFO thin films show narrow ferroelectric P-E hysteresis loops, corresponding to a soft ferroelectric. In summary, the measured properties of BZFO thin films qualify them for memory and photovoltaic device applications.

## Figures and Tables

**Table 1:** Lattice constant, bond length, and dislocation density of BFO, BDFO, and BZFO thin films.

Thin Films	Lattice Constant (Å)		Bi-O (Ả)	Fe-O (Å)	Dislocation Density ×10^−4^	Tolerance Factor
	a	c				
BFO	5.7038	13.565	2.5475	1.7868	7.71	0.7640
BDFO	5.756	13.794	2.5271	1.821	5.38	0.7632
BZFO	5.596	13.755	2.4877	1.8642	4.52	0.7624

**Table 2:** Magnetic properties of BFO, BDFO, and BZFO thin films.

Thin Films	Saturation Magnetization (emu/g)	Coercive Field (Oe)	Remnant Magnetization (emu/g)
BFO	15,053.54	224.705	0.07048
BDFO	14,806.425	233.53	39,163.6346
BZFO	641,777.53	221.442	1,014,534.83

**Table 3:** The ferromagnetic and ferroelectric values of previous reports.

Samples	Saturation Magnetization (emu/g)	Coercive Field (Oe)	Remnant Magnetization (emu/g)	Remnant Polarization (C/cm^2^)	Reference
Bi_0.95_Dy_0.05_FeO_3_			0.0314		[40]
BiFe_0.97_Zr_0.03_O_3_	9.33	28.577	0.229		[41]
BiFe_0.90_Zr_0.10_O_3_	8.14	17.192	0.143		[41]
Bi_0.9_La_0.1_Fe_0.98_Zr_0.02_O_3_			0.013		[42]
BiFe_0.90_Zr_0.05_O_3_	48				[43]
BiFe_0.90_Zr_0.10_O_3_	52.1				[44]
BiFeO_3_		113	0.12		[45]
Bi_0.90_Dy_0.10_FeO_3_				0.32	[46]
BiFe_1−2x_Zr_x_Mg_x_O_3_ (x = 0.05)	0.649	152.5	0.108		[47]

**Table 4:** Ferroelectric properties of BFO, BDFO, and BZFO thin films.

Thin Films	Coercive Field (kV/cm)	Remnant Polarization (μC/cm^2^)
BZFO	2.958	0.15589
BDFO	5.7966	0.25617
BFO	1.47564	0.20475

## Data Availability

The data used to support the findings of this study are available from the corresponding authors upon request.
